# Impact of coronavirus disease 2019 (COVID-19) on US Hospitals and Patients, April–July 2020

**DOI:** 10.1017/ice.2021.69

**Published:** 2021-02-19

**Authors:** Mathew R. P. Sapiano, Margaret A. Dudeck, Minn Soe, Jonathan R. Edwards, Erin N. O’Leary, Hsiu Wu, Katherine Allen-Bridson, Agasha Amor, Rashad Arcement, Sheri Chernetsky Tejedor, Ray Dantes, Cindy Gross, Kathryn Haass, Rebecca Konnor, Seth R. Kroop, Denise Leaptrot, Kent Lemoine, Allan Nkwata, Kelly Peterson, Lauren Wattenmaker, Lindsey M. Weiner-Lastinger, Daniel Pollock, Andrea L. Benin

**Affiliations:** 1 Division of Healthcare Quality Promotion, Centers for Disease Control and Prevention, Atlanta, Georgia; 2 Lantana Consulting Group, Thetford, Vermont; 3 Division of Hospital Medicine, Department of Medicine, Emory University School of Medicine, Atlanta, Georgia; 4 Department of Biomedical Informatics, Emory University School of Medicine, Atlanta, Georgia; 5 CACI, Atlanta, Georgia; 6 COVID-19 Response, Centers for Disease Control and Prevention, Atlanta, Georgia; 7 Leidos, Atlanta, Georgia

## Abstract

**Objective:**

The rapid spread of severe acute respiratory coronavirus virus 2 (SARS-CoV-2) throughout key regions of the United States in early 2020 placed a premium on timely, national surveillance of hospital patient censuses. To meet that need, the Centers for Disease Control and Prevention’s National Healthcare Safety Network (NHSN), the nation’s largest hospital surveillance system, launched a module for collecting hospital coronavirus disease 2019 (COVID-19) data. We present time-series estimates of the critical hospital capacity indicators from April 1 to July 14, 2020.

**Design:**

From March 27 to July 14, 2020, the NHSN collected daily data on hospital bed occupancy, number of hospitalized patients with COVID-19, and the availability and/or use of mechanical ventilators. Time series were constructed using multiple imputation and survey weighting to allow near–real-time daily national and state estimates to be computed.

**Results:**

During the pandemic’s April peak in the United States, among an estimated 431,000 total inpatients, 84,000 (19%) had COVID-19. Although the number of inpatients with COVID-19 decreased from April to July, the proportion of occupied inpatient beds increased steadily. COVID-19 hospitalizations increased from mid-June in the South and Southwest regions after stay-at-home restrictions were eased. The proportion of inpatients with COVID-19 on ventilators decreased from April to July.

**Conclusions:**

The NHSN hospital capacity estimates served as important, near–real-time indicators of the pandemic’s magnitude, spread, and impact, providing quantitative guidance for the public health response. Use of the estimates detected the rise of hospitalizations in specific geographic regions in June after they declined from a peak in April. Patient outcomes appeared to improve from early April to mid-July.

In March 2020, widespread transmission in the United States of severe acute respiratory coronavirus virus 2 (SARS-CoV-2), the virus that causes coronavirus disease 2019 (COVID-19), became apparent.^1^ The pandemic’s prior toll in China and Italy suggested that COVID-19 would stress the capacity of the US healthcare system.^2–6^ This possibility prompted the Centers for Disease Control and Prevention to extend its hospital surveillance coverage through the National Healthcare Safety Network (NHSN) to include daily inpatient censuses, COVID-19 hospitalizations, and the availability and use of ventilators and other healthcare resources.^7,8^

On March 27, 2020, the NHSN introduced a hospital COVID-19 module, a launch that was supported by the White House Coronavirus Task Force.^9,10^ This module enabled hospitals to report daily counts of patients with suspected or confirmed COVID-19 diagnoses and current use of hospital beds and mechanical ventilators. Expansion of the system on April 14 added data collection on shortages of healthcare workers and supplies.

Shortly after the hospital COVID-19 module was introduced, the NHSN program team began preparing daily reports for use in the pandemic response that included crude occupancy rates, COVID-19 hospitalizations, and ventilator use. Although these initial measures were informative, they did not account for variations in the frequency of reporting or for missing data. To account for hospitals that did not report and for missing data, the NHSN team developed weighted national and state time-series estimates. The goals for these estimates were to allow ready comparison of COVID-19 hospital indicators among states and to provide a better evaluation of the magnitude of the pandemic for public health officials and for the American public. Here, we describe the impact of COVID-19 on US inpatients and hospital capacity in the early stage (April 1 through July 14, 2020) of the pandemic using time-series estimates of the critical hospital indicators developed by NHSN to characterize the pandemic in near real time.

## Methods

### About the NHSN

The NHSN is the nation’s most comprehensive and widely used surveillance system for healthcare quality measurement and improvement.^11^ Nearly 40,000 healthcare facilities report regularly to the NHSN, including most hospitals in the United States. All hospitals receiving payment from the Centers for Medicare & Medicaid Services (CMS) are required to report healthcare-associated infection (HAI) data to the NHSN Patient Safety (PS) Component in accordance with CMS reporting rules and state reporting mandates.^12–14^ The NHSN application is developed and maintained by a diverse set of subject-matter experts with decades of experience in infection prevention, epidemiology, hospital informatics, and statistics. Additionally, the NHSN has longstanding relationships with hospitals and state health departments for the purposes of accurate, timely, and standardized data collection.

### The NHSN Patient Safety Component COVID-19 Module: Patient impact and hospital capacity

On March 27, 2020, the NHSN introduced the COVID-19 module for the collection of daily patient impact and hospital capacity data from US acute-care hospitals (ACHs), critical-access hospitals (CAHs; ie, rural Medicare-participating hospitals with 25 or fewer inpatient beds that receive CAH designation from CMS^15^), long-term acute-care hospitals (LTACHs), inpatient rehabilitation facilities (IRFs), and inpatient psychiatric facilities (IPFs), children’s hospitals, women’s hospitals, women and children’s hospitals, surgical facilities, orthopedic hospitals, military hospitals, Veterans’ Administration hospitals, and oncology hospitals.

The initial surveillance form included 13 data elements that targeted the most critical pieces of information needed to assess capacity-related issues during the pandemic, including hospital bed occupancy, and availability and use of mechanical ventilators, and number of hospitalized patients with confirmed or suspected COVID-19. COVID-19–confirmed patients were those with positive COVID-19 laboratory molecular and/or viral antigen tests. (Hospitals were instructed to exclude serology testing for antibodies.^16^ Suspected COVID-19 patients were those without a positive laboratory test but with signs and symptoms compatible with COVID-19 in accordance with CDC’s Interim Public Health Guidance for Evaluating Persons Under Investigation^17^ Definitions and reporting instructions for all data elements have been described elsewhere.^16^

The data collection form was pilot-tested prior to implementation and was paired with detailed, standardized definitions. Hospitals were able to report manually or by file import, and states, hospital-associations, and hospital systems had the ability to upload data in batch submissions. State and local health departments and healthcare systems had immediate, secure access to data and dynamic reports for hospitals within their jurisdiction, and data were shared daily across the US government for the national response to the pandemic. The NHSN COVID-19 module was an approved option for reporting patient impact and capacity-related data to the Department of Health and Human Services (HHS) through July 14, 2020.^18,19^ After July 14, the HHS requested that facilities report directly to the new HHS data system.^19^

### Calculation of state and national estimates

A daily times series of key variables was constructed using multiple imputation and survey weighting adapted from the technique described by Sapiano et al^20^ with modifications to allow regular, daily estimation. A comprehensive description of the statistical methods appears in Appendix 1 (online).

**Fig. 1.**
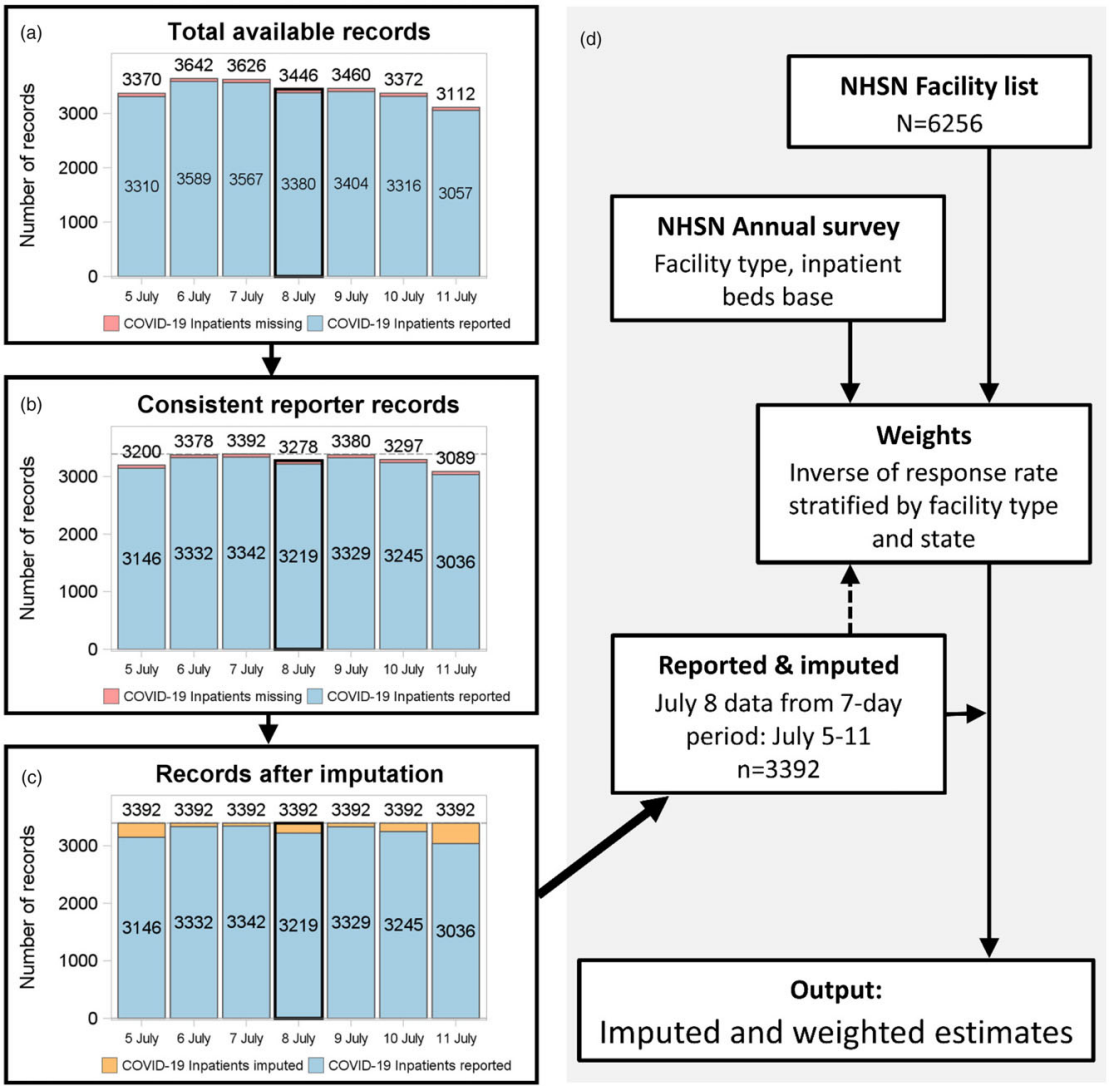
Flow-chart diagram of processing for a single example day (July 8) of estimates.

Analyses used daily data and were conducted daily. A complete hospital list, including hospital type and base number of inpatient and ICU beds, was generated based on facilities in active status. Strata for weighting and imputation used size and type of hospital. For each day, hospitals were restricted to consistent reporters with at least 5 daily records in the surrounding 7 days (Fig. 1). Missing data were imputed for the whole 7-day period for each variable using a 2-stage multiple imputation procedure for count data.^20–22^ Imputation models included the national strata, number of inpatient beds (ICU beds for ICU and ventilator variables), and the pooled mean proportion of inpatients with COVID-19 within a 50-mile radius based on ZIP code. Imputations also included the reported or imputed value for the prior day after the first day in the 7-day period.

National and state estimates for the 50 US states, Washington, DC, and Puerto Rico were computed separately for each day based on the reported and imputed (generally <5%) data for that day. National estimates were made starting April 1, 2020. State estimates started later because some states had insufficient reporting prior to April 23. The question on the number of ICU patients with COVID-19 was added on June 4, 2020. Weights were calculated as the inverse of the proportion of facilities reporting in each strata and state. Estimates for each state were calculated in a domain analysis.^23^ Confidence intervals (CIs) were estimated using the Taylor series method.^24^ Estimates and CIs for each day were compiled to produce the time series for each variable. Estimates for the last 3 days of the series were made using data imputed from the last available 7-day period in the series. This approach mirrors that used for the next-day estimation process wherein the most recent 4 days of the record were replaced each day to incorporate newly reported data after a 2–3-day reporting lag for some facilities. All analyses were carried out using SAS software version 9.4 (SAS Institute, Cary, NC). This activity was reviewed by CDC and was conducted consistent with applicable federal law and CDC policy (see eg, 45 CFR part 46, 21 CFR part 56; 42 USC §241(d); 5 USC §552a; 44 USC §3501 et seq).^2^

## Results

Table 1 summarizes hospitals reporting to the NHSN PS COVID-19 Module as well as active reporters based on the NHSN facility list. In total, 6,256 hospitals were enrolled and active in NHSN for HAI reporting, with 4,576 hospitals (73.1%) reporting COVID-19 data at least once on or prior to July 14, 2020. The number of consistently reporting hospitals (reporting an average of 5 of 7 days over the period) was 2,171 (34.7%) in April but was higher in May at 3,579 (57.2%) and June–July (combined because data end in mid-July) at 3,382 (54.1%). When split by facility type, consistent reporting was strongest among ACHs with 100 or more beds, with ˜60% or more consistently reporting in May and June–July. Overall, reporting was lower among smaller hospitals including CAHs, women’s and children’s hospitals, LTACHs, and IPFs. Coverage was even between urban (metropolitan) and rural areas.^25^

**Table 1.** Facility Characteristics for Facilities Reporting to NHSN Patient Impact and Healthcare Safety COVID-19 Module and Patient Safety Component

Facility characteristic	Facilities Reporting >21 Days per Month^a^, No. (%)^b^			Facilities Reporting at Least Once, No. (%)^b^	Enrolled Facilities, No.
	April	May	June 1–July 14		
All facilities	2,171 (34.7)	3,579 (57.2)	3,382 (54.1)	4,576 (73.1)	6,256
**Facility type**					
General ACH, ≥300 beds	272 (36.4)	478 (64.0)	438 (58.6)	607 (81.3)	747
General ACH, 200–299 beds	188 (35.1)	352 (65.8)	322 (60.2)	428 (80.0)	535
General ACH, 100–199 beds	345 (38.3)	606 (67.3)	571 (63.4)	738 (81.9)	901
General ACH, 50–99 beds	271 (40.8)	406 (61.1)	392 (58.9)	518 (77.9)	665
General ACH, 1–49 beds	250 (36.5)	341 (49.8)	322 (47.0)	463 (67.6)	685
Critical access	488 (39.1)	708 (56.7)	668 (53.5)	980 (78.5)	1249
Women/Children’s	45 (32.4)	68 (48.9)	64 (46.0)	93 (66.9)	139
Long-term ACH	133 (29.6)	167 (37.1)	163 (36.2)	220 (48.9)	450
Inpatient rehabilitation	92 (24.6)	228 (61.0)	226 (60.4)	246 (65.8)	374
Inpatient psychiatric	16 (10.3)	20 (12.9)	17 (11.0)	35 (22.6)	155
Other^c^	71 (19.9)	205 (57.6)	199 (55.9)	248 (69.7)	356
**Urban (Metropolitan) or rural**					
Metropolitan, central	1276 (32.8)	2255 (58.0)	2142 (55.1)	2793 (71.8)	3889
Metropolitan, outlying	152 (35.8)	241 (56.7)	232 (54.6)	309 (72.7)	425
Rural, micropolitan	322 (37.4)	492 (57.1)	460 (53.4)	662 (76.9)	861
Rural	421 (38.9)	591 (54.7)	548 (50.7)	812 (75.1)	1081

Note. NHSN, National Health Safety Network; ACH, acute-care hospital.

a The definition of consistent reporting facilities is aligned with the definition used for the estimates, which is that facilities must have at least five of seven days reporting and includes facilities with at least 21 records in April and May and at least 31 records during June 1–July 14.

b Denominator for percentages is the number of enrolled facilities.

c Other includes surgical, orthopedic, military, Veterans’ Administration, oncology.

**Fig. 2.**
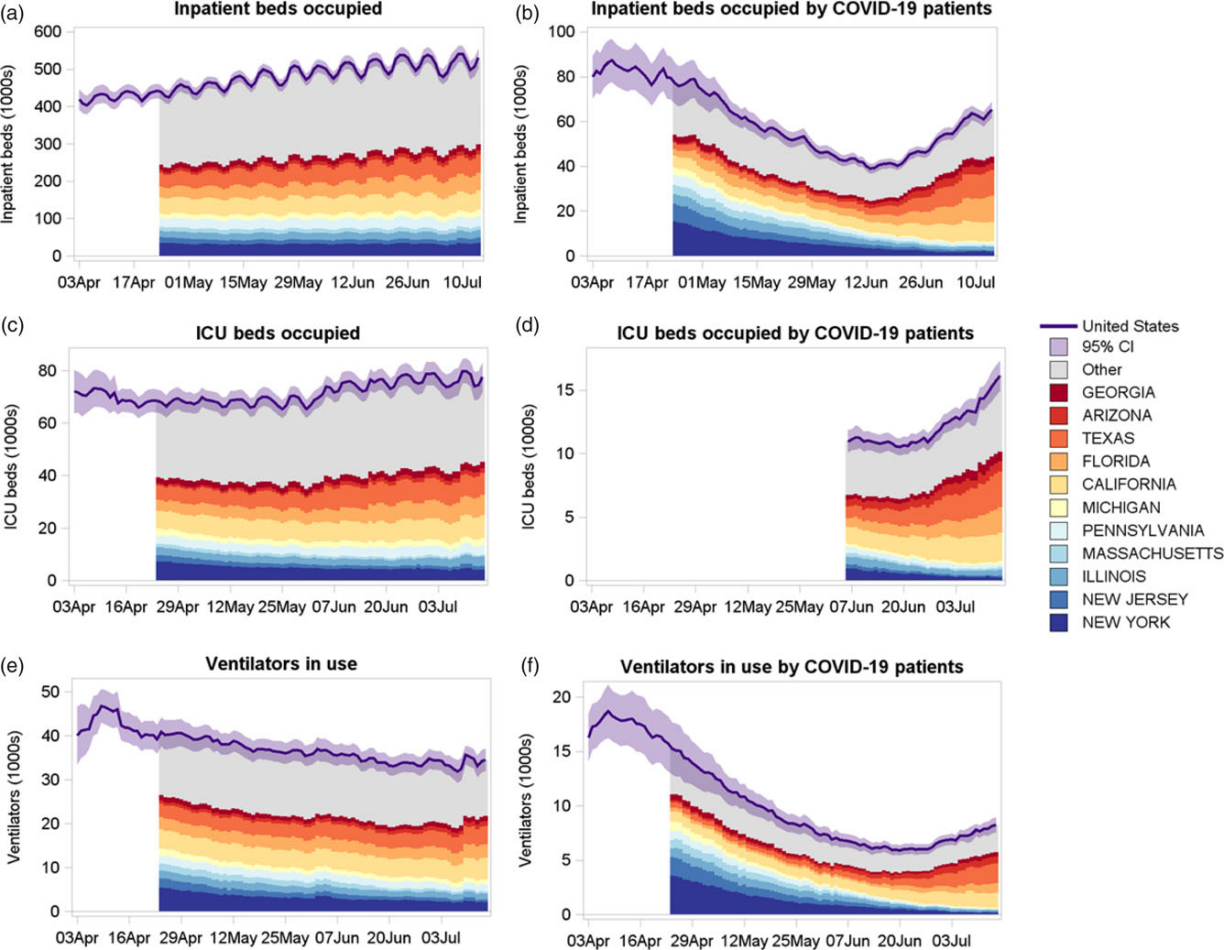
Time-series stacked bar plots showing inpatient beds occupied, inpatient beds occupied by patients with confirmed or suspected COVID-19, ICU beds occupied, ICU beds occupied by confirmed or suspected COVID-19 patients, ventilators in use and ventilators in use by patients with confirmed or suspected COVID-19 during April 1–July 14, 2020.

Figure 2 shows the daily time series of inpatient beds occupied, inpatient beds occupied by patients with COVID-19, intensive care unit (ICU) beds occupied, ICU beds occupied by patients with COVID-19, ventilators in use, and ventilators in use by patients with COVID-19 from April 1 to July 14, 2020. State estimates are presented starting April 24 due to insufficient reporting in several states prior to that date. The same day of the week was used for comparisons because of the weekly cycle in occupancy. The number of inpatient beds occupied in the United States increased steadily from 431,195 (95% CI, 407,836–454,555) on Friday, April 10 to 540,491 (95% CI, 517,890–563,092) on Friday, July 10. Over the same period, the number of inpatient beds occupied by patients with COVID-19 decreased from a national high of 84,149 (95% CI, 74,959–93,340) on Friday, April 10 to 62,880 (95% CI, 59,725–66,035) on Friday July 10, with a low around 43,158 (95% CI, 40,582–45,734) on Friday, June 5. Figure 2 also shows the contribution to the national estimate from states that had at least 5% of the hospitalizations at any point between April 24 and July 14. New York had the most daily hospitalized patients (15,517; 95% CI, 11,176–19,857) followed by New Jersey, Illinois, Massachusetts, Pennsylvania, Michigan, and California. As cases declined in these states, the number of US hospitalized cases declined correspondingly, but this number began to increase as the pandemic shifted to other states. The period when some state-based restrictions were eased in late May and early June was followed by increased hospitalizations for COVID-19 and overall hospital admission increases. During late June and early July, the increase in national inpatient beds occupied by patients with COVID-19 was driven by cases in California, Florida, Texas, Arizona, and Georgia.

Between April 24 and July 10, the national number of ICU beds occupied was somewhat stable, but contributions by state fluctuated, with New York falling from 7,456 (95% CI, 5,191–9,722) on April 24 to 4,859 (95% CI, 3,009–6,708) on July 10. The number of ventilators in use by patients with COVID-19 showed strong contrast between states at the start and the end of the period. Again, New York had the highest number of ventilated patients with COVID-19 with 3,600 (95% CI, 2,477–4,724) on April 24, but this fell substantially to 216 (95% CI, 108–323) on July 10. During the latter part of the period, increases occurred in ventilated patients with COVID-19 in Texas (n = 1,603 on July 10; 95% CI, 1,238–1,969), California (n = 1,271; 95% CI, 1,067–1,476), Florida (n = 749; 95% CI, 542–957), Arizona (n = 610; 95% CI, 303–916), and Georgia (n = 414; 95% CI, 247–582).

Table 2 shows inpatient and ICU bed occupancy and ventilator use by hospital type. The ACH group represented the largest share of occupancy, with 76.3% of inpatients admitted to an ACH with 100 or more beds on Friday, April 24. These larger hospitals saw a disproportionately higher percentage of inpatients with COVID-19 with 88.1% of all inpatients with COVID-19 admitted to an ACH with 100 or more beds and 94.1% of ventilators in use by patients with COVID-19 in ACHs with 100 or more beds. CAHs account for 1,249 of 6,256 hospitals reporting to the module and are situated in rural and underserved areas. CAHs accounted for a small percentage of inpatients (1.9% on April 24) and a smaller proportion of inpatients with COVID-19 (1.0%). Other hospital types accounted for 14.6% of all inpatient beds occupied but only 4.1% of inpatients with COVID-19. These other hospitals accounted for 18.1% of all ventilators in use but only 1.0% of ventilators in use by patients with COVID-19.

**Table 2.** Inpatient and ICU Bed Occupancy and Ventilator Use for All Patients and for Patients With Confirmed or Suspected COVID-19 Stratified by Facility Type for Friday, April 24, 2020, and Friday, July 10, 2020

		No. (% of National Estimate; 95% CI)					
Date	Facility Type	Inpatient Beds Occupied	Inpatient Beds Occupied by Patients With COVID-19	ICU Beds Occupied	ICU Beds Occupied by Patients With COVID-19	Ventilators in Use	Ventilators in Use by Patients With COVID-19
April 24, 2020	General ACH, 300 or more beds	198,828 (45.3%; 181,557–216,099)	40,737 (52.7%; 31,963–49,511)	36,360 (53.2%; 32,484–40,235)		20,711 (50.7%; 17,763–23,659)	9,279 (60.9%; 6,947–11,611)
	General ACH, 200–299 beds	68,780 (15.7%; 64,823–72,737)	13,480 (17.5%; 11,589–15,371)	9,877 (14.5%; 9,121–10,633)		5,490 (13.4%; 4,911–6,069)	2,480 (16.3%; 2,108–2,852)
	General ACH, 100–199 beds	67,145 (15.3%; 64,065–70,225)	13,830 (17.9%; 12,126–15,534)	9,862 (14.4%; 9,210–10,515)		5,608 (13.7%; 5,021–6,196)	2,569 (16.9%; 2,189–2,949)
	General ACH, 50–99 beds	22,646 (5.2%; 21,054–24,238)	3,761 (4.9%; 3,135–4,388)	3,449 (5.0%; 3,119–3,780)		1,269 (3.1%; 1,035–1,502)	611 (4.0%; 475–747)
	General ACH, 1–49 beds	8,641 (2.0%; 7,874–9,408)	1,467 (1.9%; 1,124–1,809)	1,142 (1.7%; 953–1,330)		330 (0.8%; 221–439)	130 (0.9%; 41–219)
	Critical Access	8,312 (1.9%; 7,694–8,931)	802 (1.0%; 645–959)	310 (0.5%; 237–383)		75 (0.2%; 21–129)	16 (0.1%; 0–39)
	Other^a^	64,239 (14.6%; 58,129–70,349)	3,159 (4.1%; 2,012–4,307)	7,312 (10.7%; 5,385–9,239)		7,398 (18.1%; 6,082–8,713)	157 (1.0%; 75–238)
July 10–2020	General ACH, 300 or more beds	249,341 (46.1%; 229,696–268,986)	28,106 (44.7%; 25,676–30,536)	42,699 (53.5%; 38,787–46,612)	6,967 (48.6%; 6,180–7,755)	17,394 (49.4%; 15,691–19,096)	3,978 (52.2%; 3,522–4,433)
	General ACH, 200–299 beds	83,623 (15.5%; 77,400–89,845)	11,748 (18.7%; 10,463–13,032)	10,793 (13.5%; 9,738–11,849)	2,450 (17.1%; 2,122–2,778)	4,792 (13.6%; 4,260–5,323)	1,431 (18.8%; 1,197–1,665)
	General ACH, 100–199 beds	81,431 (15.1%; 76,743–86,119)	11,986 (19.1%; 10,878–13,095)	10,150 (12.7%; 9,393–10,907)	2,852 (19.9%; 2,515–3,188)	4,113 (11.7%; 3,695–4,531)	1,382 (18.1%; 1,164–1,599)
	General ACH, 50–99 beds	26,558 (4.9%; 24,617–28,499)	3,943 (6.3%; 3,319–4,566)	3,505 (4.4%; 3,177–3,833)	1,035 (7.2%; 826–1,243)	1,224 (3.5%; 1,000–1,448)	387 (5.1%; 290–484)
	General ACH, 1–49 beds	10,342 (1.9%; 9,356–11,327)	1,720 (2.7%; 1,376–2,065)	1,238 (1.5%; 1,019–1,457)	487 (3.4%; 337–636)	319 (0.9%; 221–417)	83 (1.1%; 31–136)
	Critical Access	9,759 (1.8%; 9,015–10,503)	567 (0.9%; 439–696)	421 (0.5%; 323–518)	63 (0.4%; 34–91)	57 (0.2%; 18–96)	12 (0.2%; 0–35)
	Other*	79,438 (14.7%; 71,920–86,956)	4,809 (7.6%; 4,002–5,616)	11,071 (13.9%; 8,677–13,465)	495 (3.4%; 253–736)	7,322 (20.8%; 6,060–8,584)	349 (4.6%; 257–441)

Note. CI, confidence interval; ICU, intensive care unit; ACH, acute-care hospital.

a Other facility types include women/children’s, long-term ACH, inpatient rehabilitation, inpatient psychiatric, surgical, orthopedic, military, Veterans’ Administration, oncology.

**Fig. 3.**
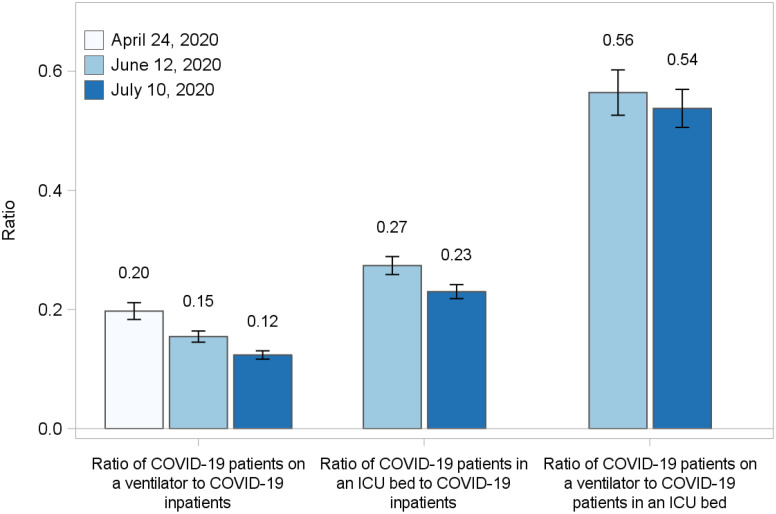
Bar charts comparing ratios of patients with confirmed or suspected COVID-19 on a ventilator to inpatients with confirmed or suspected COVID-19, patients with confirmed or suspected COVID-19 in an ICU bed to inpatients with confirmed or suspected COVID-19, and patients with confirmed or suspected COVID-19 on a ventilator to patients with confirmed or suspected COVID-19 in an ICU bed for Friday April 24, Friday June 12, and Friday July 10, 2020, with 95% confidence intervals.

The ratios of patients on ventilators and in the ICU to all inpatients may be used as an indicator of patient severity. Figure 3 shows the ratio of patients with COVID-19 on a ventilator to inpatients with COVID-19. The ratio fell from 0.20 (95% CI, 0.18–0.21) on April 24 to 0.12 (95% CI, 0.12–0.13) on July 10, suggesting that the number of ventilated patients with COVID-19 compared to the number of inpatients with COVID-19 was lower in mid-July than in late April. A similar decrease was evident in the ratio of patients with COVID-19 in an ICU bed to all inpatients with COVID-19, which dropped from 0.27 (95% CI, 0.26–0.29) on June 12 to 0.23 (95% CI, 0.22–0.24) on July 10. The ratio of patients with COVID-19 on a ventilator to patients with COVID-19 in an ICU bed was unchanged from 0.56 (95% CI, 0.53–0.60) on June 12 to 0.54 (95% CI, 0.51–0.57) on July 10.

## Discussion

At the first peak of the pandemic in the United States around April 10, 2020, the NHSN estimated 84,000 inpatients with suspected or confirmed COVID-19 among 431,000 total inpatients. After the April peak of inpatients with COVID-19, overall inpatient bed occupancy continued to increase. The creation of regular, daily estimates allowed public health personnel to monitor and respond to the rising numbers of inpatients that began in the middle of June and quickly overwhelmed hospitals in the South and Southwest regions, driven by increases in Florida, Texas, Arizona, and Georgia. These estimates were created 3 times weekly to update data to the previous day and were disseminated within the government, where they were used for prioritization of staff deployments, model calibration and validation, the creation of hospital capacity thresholds, and in decisions affecting personal protective equipment and remdesivir allocations to hospitals. In addition, the estimates were disseminated to state health departments 3 times weekly as part of a package of hospital capacity data, and daily data were released to the general public on a weekly basis via the CDC website.

Existing strong relationships with hospitals, hospital associations, and states forged over decades of hospital surveillance, supported the successful rapid activation of the NHSN to create a vendor-neutral, unified national system for hospital surveillance information during the early stages of the COVID-19 pandemic in the United States. The NHSN provided the first national- and state-level lens into the burden of disease in hospitals and hospitals’ capacity to respond. In addition, the rapid development and implementation of new data collection protocols and systems, the NHSN also developed standardized definitions of hospital capacity metrics that have remained the standard for COVID-19 surveillance. Prior to this work, lack of standardization of such metrics resulted in an inconsistent understanding of hospital capacity across the nation.

The information resulting from the NHSN COVID-19 module had 2 major elements. The first element concerns the level of care required for patients with COVID-19. The ratio of patients with COVID-19 on a ventilator to all inpatients with COVID-19 decreased between April and mid-July, and the ratio of patients with COVID-19 in an ICU bed to inpatients with COVID-19 also decreased from mid-June to mid-July, with relatively fewer patients requiring treatment in the ICU or ventilation. This downward trend is consistent with improvements in treatment for COVID-19,^26–29^ but it may also indicate that patients admitted in late June and July had lower risk of severe complications associated with comorbidities and age among other potential factors. The ratio of patients with COVID-19 on a ventilator to patients with COVID-19 in an ICU bed was relatively unchanged between mid-June and mid-July, suggesting that the need for mechanical ventilation among the sickest patients was unchanged over this shorter period.

The second element was hospital capacity, which was a major concern for the pandemic response. Initially, many hospitals postponed elective admissions and emergency department visits decreased.^30,31^ As the pandemic continued into June and July of 2020, hospital occupancy increased nationally; at the same time, the outbreak progressed into regions that had lifted stay-at-home orders. The highest burden for treatment of patients with COVID-19 fell on ACHs with >100 beds that treated 88.1% of all inpatients with confirmed or suspected COVID-19 and 94.1% of all ventilators in use by patients with COVID-19 on April 24, 2020. Although smaller ACHs and CAHs had relatively small proportions of patients with COVID-19, tracking the impact in these facilities was important for the allocation of therapeutics and supplies, particularly for CAHs that may be the only practical option for underserved rural populations.

This study has several limitations. The data set was intended for regular, daily operational use, and our intent was to describe this operational data set and results as a basis for future near–real-time monitoring. However, minor discontinuities may remain in the series, and interpretation of variations below the level of statistical confidence are not recommended. The source data and estimates have not been validated. Data on the number of ICU patients with COVID-19 were not available until June 4, 2020, resulting in a shorter time series. Data were self-reported by facilities. The statistical method did not include time information, which could improve the estimates and reduce uncertainty.

These results provide important lessons for COVID-19 hospital surveillance during the continuing pandemic, as well as for future pandemics and outbreaks. The approach described is based on existing survey methods, and the extension to this arena lends a powerful tool for pandemic and other surveillance in the absence of complete reporting. This approach should be considered for other metrics within the hospital surveillance sphere, as well as to other areas of surveillance of hospital-based metrics. Accurate national and state estimates require high-quality, facility-level, daily data with limited missing elements. Such enhancements should be considered for systems for surveillance of hospital-based indicators. Although the present analysis highlights hospital burden during the pandemic, important work remains to quantify the impact of the pandemic on routine hospital operations, especially on healthcare-associated infections that are routinely tracked by the NHSN. This remains an important area of ongoing work for the CDC.

## Supplementary material

For supplementary material accompanying this paper visit https://doi.org/10.1017/ice.2021.69. click here to view supplementary material
