# Supplementary material for: Impact of coronavirus disease 2019 (COVID-19) on US Hospitals and Patients, April–July 2020
Source: Infect Control Hosp Epidemiol. 2021 Feb 19:1–8. doi: 10.1017/ice.2021.69 (PMC7943952; doi:10.1017/ice.2021.69)
Supplement: Supplementary file 1 [file S0899823X21000696sup001.docx]

**Supplementary material**

**Contents:**

Table S1. NHSN Patient Safety Component COVID-19 Module: Definitions for Select Variables and Metrics. Page 2.

Appendix: Statistical methods for the near real time production of national and state estimates of COVID-19 hospitalization. Page 3.

Mathew R. P. Sapiano, PhD^1,2^, Margaret A. Dudeck, MPH^1^, Minn Soe, MBBS^1^, Jonathan R. Edwards, MStat^1^, Erin N. O’Leary, MPH^1,2^, Hsiu Wu, MD^1^, Katherine Allen-Bridson, MScPH^1^, Agasha Amor, MPH^2^, Rashad Arcement, MSPH^1,5^, Sheri Chernetsky Tejedor, MD^1,3,4^, Ray Dantes, MD^1,3^, Cindy Gross, BS^1,5^, Kathryn Haass, MPH^1^, Rebecca Konnor, MPH^1,5^, Seth R. Kroop, MPA^6^, Denise Leaptrot, MSA^1,5^, Kent Lemoine, MBA^1^, Allan Nkwata, MPH^1,7^, Kelly Peterson, BBA^1^, Lauren Wattenmaker, MPH^1^, Lindsey M. Weiner-Lastinger MPH^1^, Daniel Pollock, MD^1^, Andrea L. Benin, MD^1^ and the NHSN Hospital COVID-19 Team

^1^ Division of Healthcare Quality Promotion, Centers for Disease Control and Prevention

^2^ Lantana Consulting Group, Inc

^3^ Division of Hospital Medicine, Department of Medicine, Emory University School of Medicine

^4^ Department of Biomedical Informatics, Emory University School of Medicine

^5^ CACI, Atlanta, GA

^6^ CDC COVID-19 Response

^7^ Leidos, Atlanta, GA

Disclaimer: The findings and conclusions in this article are those of the authors and do not necessarily represent the official position of the U.S. Centers for Disease Control and Prevention. The use of trade names is for identification purposes only and does not constitute endorsement by the U.S. Centers for Disease Control and Prevention or the Department of Health and Human Services.

**TABLE S1**. NHSN Patient Safety Component COVID-19 Module: Definitions for Select Variables and Metrics.

| **Description** | **Type** | **Definition** | **Notes** |
| --- | --- | --- | --- |
| Facility type | Count | Type of inpatient healthcare facility reporting, based on facility’s self-identified NHSN enrollment information. |  |
| Inpatient beds | Count | Total number of inpatient beds set up and staffed. | Base values from NHSN patient safety annual survey |
| Inpatient beds occupied | Count | Total number of inpatient beds occupied by any patient. |  |
| ICU beds | Count | Total number of intensive care unit (ICU) beds set up and staffed. | Base values from NHSN patient safety annual survey |
| ICU beds occupied | Count | Total number of ICU beds occupied by any patient. |  |
| Ventilators available | Count | Total number of mechanical ventilators available. | Missing values were filled by propagating last entered value |
| Ventilators in use | Count | Total number of mechanical ventilators in use by any patient. |  |
| Hospitalized COVID-19 patients | Count | Total number of suspected or confirmed COVID-19 patients hospitalized in an inpatient bed. |  |
| Hospitalized ICU COVID-19 patients | Count | Total number of suspected or confirmed COVID-19 patients hospitalized in an ICU bed. |  |
| Hospitalized COVID-19 patients on a ventilator | Count | Total number of suspected or confirmed hospitalized COVID-19 patients on mechanical ventilation. |  |

**Appendix: Statistical methods for the near real time production of national and state estimates of COVID-19 hospitalization.**

The following describes the approach used to create near-real time state and national estimates for the National Healthcare Safety Network (NHSN) Patient Safety COVID-19 Module during April 1–July 14 in more complete detail.

Prior to estimation, a list of facilities that were enrolled in the NHSN Patient Safety module that were deemed as active reporters was generated; facilities that had closed were excluded. The facility list included the hospital type (i.e. general acute care, critical access, inpatient rehabilitation facility, women and children’s, long-term acute care, inpatient psychiatric, surgical, orthopedic, military, Veterans Administration, and oncology ) and included the number of inpatient and ICU beds from the most recent NHSN annual survey. Many facilities updated reported ventilator value at less than daily frequency, and the last known value was propagated forward until an updated value was entered. Strata for weighting and imputation were constructed using facility type and size information. General Acute Care Hospitals were divided into five strata based on number of inpatient beds (1–49 beds, 50–99 beds, 100–199 beds, 200–299 beds and 300 or more beds), with the rest of the facilities divided into Critical Access Hospitals and other (includes any active reporting women/children’s, long-term acute care, inpatient rehabilitation, inpatient psychiatric, surgical, orthopedic, military, Veterans Administration, and oncology). Finally, NHSN includes quality control during the data entry phase based on a set of standard business rules, such as upper and lower bounds on input values. Additional quality control was applied prior to imputation including the removal of values within each stratum exceeding seven standard deviations from the mean were set to missing.

Figure 1 shows the processing flow for an example day (July 8). Reporting was not mandatory, and a small number of reporting hospitals did not report consistently. This added the potential for discontinuities in the time series, so consistent reporting facilities were defined using a seven-day window centered on the day of interest. Facilities with reported data for at least five of seven days were considered consistent reporters. After the data were restricted to consistent reporters, missing data (missing data items and completely missing records) were imputed for the whole seven-day period so that each day had the same number of records. Missing data were imputed using multiple imputation following the approach described by Sapiano et al^1^ for count data^2^. A two-stage multiple imputation procedure^3^ was used to impute first the occurrence (using a logistic regression model), and then the magnitude (using a log-normal regression model) for observations that were imputed as non-zero in the first stage. Each observation was imputed 20 times. The imputation models included the national strata, number of inpatient beds (ICU beds for ICU variables and ventilator variables) and the pooled mean proportion of inpatients with COVID-19 within a 50-mile radius. In addition, the number of ventilators was also included in the model for the imputation of ventilator estimates. The pooled mean proportion of inpatients with COVID-19 within a 50-mile radius was calculated for each facility based on all reporting facilities (excluding the target facility) within a 50-mile radius, or the proportion at the nearest facility if there were no reporting facilities within 50 miles. Distances were calculated using the great-circle distance based on the coordinates of the facility zip code centroid. Finally, after the first day in the 7-day period, the reported or imputed value for the prior day was included.

National and state estimates for the 50 U.S. states, Washington D.C. and Puerto Rico, were computed separately for each day based on the reported data and the imputed data (generally less than 5%) for that day. Weights were calculated as the inverse of the proportion of facilities reporting in each strata and state. State estimates were calculated in a domain analysis^4^. Count data were estimated as the weighted sum and ratio metrics were estimated as the weighted ratio. Confidence intervals (CI) for the weighted sums and weighted ratios were estimated using the Taylor series method^5^. Estimates (and CIs) for each day were compiled to produce the time series for each variable. Data for the analysis shown were reprocessed to include all data for entered up to July 14, 2020.

No data were collected after July 14, 2020, so estimates for the last three days (July 12, 13, 14) were made using data imputed from the last available seven-day period in the series (July 8–14) with weights from this same period. This approach mirrors that used for the near-real time estimate. Data reported to the NHSN Patient Safety COVID-19 module typically had a reporting lag, with approximately half of all daily reports were reported the next day, with the other half reported two or three days later. In order to provide near-real time, next-day estimates the most recent three days were constructed using the most recent seven-day period. For example, data for COVID-19 inpatients for July 14 were estimated using the 3079 reported and imputed records (1938 reported data items and 1141 imputed data items) with the same weights as were used for July 11 (fig. 1). The most recent four days were therefore updated each day and estimates were only finalized once they reached four days old.

This paper focuses on a subset of the multiple imputed, weighted variables. Specifically, national and state estimates are presented based on data reported to NHSN for inpatient beds occupied, inpatient beds occupied by COVID-19 patients, ICU beds occupied, ICU beds occupied by COVID-19 patients, ventilators in use, and ventilators in use by COVID-19 patients. The number of inpatient and ICU beds excluded from the estimation method because both were known for all facilities. The number of inpatient and ICU beds were calculated using the number reported in the NHSN Patient Safety COVID-19 Module or the base number of inpatient or ICU beds from the most recent annual facility survey. All analyses were carried out using SAS software version 9.4 (SAS Institute Inc., Cary, NC, USA).

REFERENCES

1. Sapiano MRP, Savinkina AA, Ellingson KD, et al. Supplemental findings from the National Blood Collection and Utilization Surveys, 2013 and 2015. 2017;57:1599-624.

2. Rubin DB. Multiple Imputation for Nonresponse in Surveys. New York: Wiley; 1987.

3. He Y, Raghunathan TE. Tukey's gh Distribution for Multiple Imputation. The American Statistician 2006;60:251-6.

4. Lewis TH. Complex Survey Data Analysis with SAS. 1st edition ed. Boca Raton, FL: CRC Press; 2016.

5. Woodruff RS. A Simple Method for Approximating the Variance of a Complicated Estimate. Journal of the American Statistical Association 1971;66:411-4.
